# Preprocessing the Nintendo Wii Board Signal to Derive More Accurate Descriptors of Statokinesigrams

**DOI:** 10.3390/s16081208

**Published:** 2016-08-01

**Authors:** Julien Audiffren, Emile Contal

**Affiliations:** 1 CMLA, ENS Cachan, CNRS, Université Paris-Saclay, 94235 Cachan, France; secretariat@cmla.ens-cachan.fr; 2 Cognac-G, CNRS, Université Paris Descartes, 75006 Paris, France

**Keywords:** wii balance board, balance, postural sway, resampling, non uniform acquisition frequency

## Abstract

During the past few years, the Nintendo Wii Balance Board (WBB) has been used in postural control research as an affordable but less reliable replacement for laboratory grade force platforms. However, the WBB suffers some limitations, such as a lower accuracy and an inconsistent sampling rate. In this study, we focus on the latter, namely the non uniform acquisition frequency. We show that this problem, combined with the poor signal to noise ratio of the WBB, can drastically decrease the quality of the obtained information if not handled properly. We propose a new resampling method, Sliding Window Average with Relevance Interval Interpolation (SWARII), specifically designed with the WBB in mind, for which we provide an open source implementation. We compare it with several existing methods commonly used in postural control, both on synthetic and experimental data. The results show that some methods, such as linear and piecewise constant interpolations should definitely be avoided, particularly when the resulting signal is differentiated, which is necessary to estimate speed, an important feature in postural control. Other methods, such as averaging on sliding windows or SWARII, perform significantly better on synthetic dataset, and produce results more similar to the laboratory-grade AMTI force plate (AFP) during experiments. Those methods should be preferred when resampling data collected from a WBB.

## 1. Introduction

The Wii Balance Board (WBB) was released in 2007 by Nintendo as an accessory for the Wii game console. The WBB is a force platform, with one pressure sensor at each of its corners. Its was designed to measure the displacement of the Center of Pressure (CoP) of a person standing on the platform. The CoP is defined as the projection on the ground plane of the centroid of the vertical force distribution, and is often considered as an approximation of the vertical projection of the center of gravity, although this assumption holds true only when the body is static [1]. Although its intended application was video games, the WBB quickly gained significant attention as a tool for rehabilitation (see, e.g., [2]), and later as a device to evaluate balance and quantify of the postural control in humans in Posturography (see, e.g., [3]).

Maintaining balance is a complex control task, and postural control is a complex process which involves the integration of much sensory information, such as visual, vestibular, and proprioceptive, together with the reliance on the passive properties of the musculoskeletal system [4]. The main consequences of dysfunctions of postural control are falls, which are the second cause of accidental deaths worldwide [5]. The elderly are particularly vulnerable to falls (see, e.g., [6,7]), regarding both the prevalence and the gravity of falls. For these reasons, an early detection of the shortcomings of postural control is important, as specific actions and preventive health care (see, e.g., [8,9]) can help to prevent falls and its damages.

One of the most common way to evaluate the postural control in Posturography is through the record of a statokinesigram, i.e., the trajectory of the CoP, of a patient during a test, using one or two laboratory grade force-platforms. Those devices are expensive and generally difficult to transport, and thus are not generally available outside of specialized clinics. Since the WBB can also record statokinesigrams, and is affordable and portable, it has been proposed as an alternative to the expensive high-end force platforms (see, e.g., [3] ). Despite the increasing popularity of the WBB in Posturography, (see, e.g., [10]), many researchers have argued against the use of the WBB for postural control assessment (see, e.g., [11]). We refer the viewer to [12] for a more in depth review of this debate.

The main shortcomings of the WBB are its sub-par accuracy, its inconsistent sample rate and its poor signal to noise ratio, which makes the WBB an unreliable out-of-the-box device to measure postural control. Recently, [12] have proposed a new method for calibrating the WBB which leads to a significantly improved accuracy. They compared the signals of the WBB and the AMTI using an inverted pendulum mechanical system, and computed a linear model to retrieve the AMTI signal from the WBB signal using linear regression. The results of their work are promising and the calibrated WBB is shown to be much more accurate, and closer to laboratory grade force platforms.

However, to this day, the problem of the inconsistent sampling of the WBB has been neglected. Combined with the poor signal to noise ratio, this problem can lead to very poor results when not handled properly. As discussed in Section 2.1, many works in Posturography do not acknowledge this problem, and by artificially forcing a constant sampling rate, they use, perhaps unknowingly, a piecewise constant interpolation, which leads to greatly suboptimal results (see Section 3.1). This problem is magnified when differentiating the signal, which is necessary to compute the speed of the CoP. Since this indicator is one of the main quantifiers of postural control (see, e.g., [13]), the use of a correct resampling method is of paramount importance.

In this study, we discuss the non-uniform sampling limitation of the WBB. We then present and study several resampling approaches used in the Posturography literature to derive a uniformly sampled signal. Additionally, we introduce a new resampling method, SWARII, specifically designed with the WBB in mind, for which we provide an open source implementation. We compare those different methods by applying them to three different datasets: (1) a synthetic dataset, whose parameters were chosen to highlight the shortcoming of the most commomly used resampling approaches; (2) a WBB dataset obtained by recording immobile objects; and (3) a comparison dataset, where 10 subjects have been recorded using both the WBB and the AMTI force platform.

## 2. Materials and Methods

In this section we first introduce the non uniform sampling problem of the Wii Board Balance (Section 2.1), then we discuss the resampling method commonly used in the Posturography literature (Section 2.2) and their limitation. In Section 2.3, we introduce a new algorithm, Sliding Window Average with Relevance Interval Interpolation (SWARII), designed to better apprehend the non constant time frequency acquisition of the WBB. Finally, we describe experiments that we conducted both on simulated and real dataset ( Section 2.4 and Section 2.5) in order to compare the different resampling algorithms, and we discuss their respective analysis.

### 2.1. The WBB

**Figure 1:**
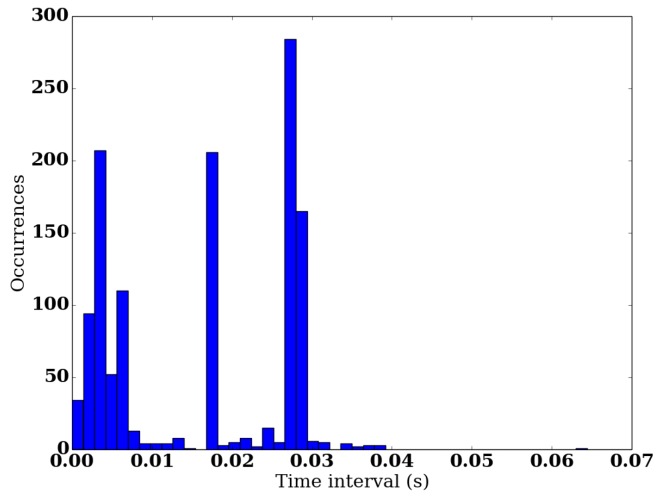
Repartition of time intervals between two consecutive data points recorded by the Wii Balance Board (WBB) during a 20 s record. The distribution is concentrated around 1 to 5 ms, 18 ms and 28 ms.

In previous studies, the WBB was reported to have different frequencies of acquisition, such as 100 Hz in [14,15], 40 Hz in [3] or 30 Hz in [16]. However, as mentioned in [11], the actual average sampling frequency of the WBB is around 63 Hz, and this frequency is not constant and varies significantly throughout an acquisition. As shown in Figure 1, during a 20 s acquisition, the WBB alternates between three different regimes: a fast paced regime (>100 Hz), a moderate regime (≈54 Hz) and a slow regime (≈35 Hz). Some points fall outside of these categories, but since they represent less than 0.1% of the total acquisition, they can be considered as outliers and safely ignored. It is important to note that the relative distribution of those regimes is constant, and is observed regardless of the actual charge of the WBB, for any sufficiently long record. In our experiments, a duration of 10 s was sufficient to observe this distribution.

As most of the existing Signal Processing tools only apply to uniformly sampled signals, it is important to pre-process the WBB signal after the acquisition. There exists several methods to resample the data, i.e., to interpolate the recorded data in order to obtain a uniformly sampled signal. Those methods include, but are not limited to, polynomial interpolation, sliding window average or spline interpolation.

However, in the WBB case, this strong variability of the acquisition frequency is combined with the poor signal to noise ratio of the WBB (see, e.g., [11]). As seen below, the lack of noise control mechanism in the resampling method can lead to significant problems during the analysis of the statokinesigrams, particularly when the signal is differentiated, which is important for computing the instantaneous speed. This aggravating phenomenon can be offset by the inclusion of a significant regularization component in the resampling method, which is the case of some of the methods presented in this section.

In the next subsection, we provide a review of some of the methods commonly used in the Posturography literature.

### 2.2. Some Common Processing Methods in Posturography

**Notation:** Let n∈N be the number of data points generated by the WBB during the record. We denote by $T=\{t_{1},\ldots ,t_{n}\}$ the set of the times at which the WBB generated those data points and $X=\{x_{t},t\in T\}$ the set of the values of the data points.

**Piecewise Constant Interpolation:** (See, e.g., [16]). In this approach the WBB is sub-sampled at a given frequency. The value of a resampled point correspond to the last recorded value by the WBB. This is equivalent to doing a Piecewise Constant Interpolation (PCI) as it assumes that the value of the signal has not changed since the last recorded value. Hence,

$$X_{PCI}\left(s\right)=x_{t^{-}}\;$$

where $X_{PCI}\left(s\right)$ is the interpolated signal at time *s* and $t^{-}=\max\left\{t\in T,t\leq s\right\}$. This approach presents a significant drawback as significant information may be lost since all the events and data points occurring between two sampling times of the WBB are discarded. This method does not include any regularisation, thus in order to reduce the noise, a Butterworth low-pass filter with a cutoff frequency of 12 Hz is used on the resulting signal.

**Linear Interpolation:** (See, e.g., [17]). In this approach in order to resample the signal, the value at the desired time *s* is obtained using a Linear Interpolation (LI) using the closest past and future points generated by the WBB, i.e.,

$$X_{LI}\left(s\right)=x_{t^{-}}\frac{t^{+}-t}{t^{+}-t^{-}}+x_{t^{+}}\frac{t-t^{-}}{t^{+}-t^{-}}\;$$

where $X_{LI}\left(s\right)$ is the interpolated signal at time *s*, $t^{-}=\max\left\{t\in T,t\leq s\right\}$ and $t^{+}=\min\left\{t\in T,t>s\right\}$. Like in the previous method, the resampled signal need to be filtered in order to reduce the impact of the noise.

**Sliding Window Average Interpolation:** (See, e.g., [12]). In this approach, the value at the desired time *s* is obtained using a Sliding Window Average Interpolation (SWAI), i.e., by averaging all the values generated by the WBB in a temporal window centered around *s* of length Δ>0:

$$X_{SWAI}\left(s\right)=\frac{1}{|T_{s}\left(\Delta \right)|}\sum_{t\in T_{s}\left(\Delta \right)}x_{t}\;$$

where $X_{SWAI}\left(s\right)$ is the interpolated signal at time *s* and $T_{s}\left(\Delta \right)=\left\{t\in T,|t-s|\leq \Delta \right\}$. Since this method relies on averaging multiple points, it includes a smoothing component, controlled by Δ, the length of the window.

**Smoothing Splines:** (See, e.g., [18]). The smoothing splines approach computes the minimizer *f* of the following regularized least-square fitting term:

$$\sum_{t\in T}{\left(x_{t}-f\left(t\right)\right)}^{2}+\lambda \int_{t_{1}}^{t_{n}}f^{\prime \prime }{\left(t\right)}^{2}dt\;$$

over the class of twice differentiable functions [19]. The solution *f* is a piecewise polynomial function. The values at the desired times are then simply obtained by evaluations of *f*. The parameter *λ* controls the smoothness of the interpolation. When λ=0, then *f* is a cubic splines with exact interpolation of the data. The former suffers from heavy overfitting in the presence of noise. When λ=∞, then *f* is the solution of a linear least-square problem. Choosing *λ* to enforce the smoothness condition is challenging in our problem since this leads to flattening the derivatives.

### 2.3. An Alternative Method for Resampling: SWARII

Although it generally gives satisfying results in practice (see, e.g., Section 3.2), one of the main limitation of the SWAI approach is that all data points in the windows are considered of equal value, which can be suboptimal in the WBB case. Indeed, if many data points are produced by the WBB in a very short interval (say e.g., 5 ms), then it is reasonable to assume that noise accounts for most of the differences between those values. Hence, by averaging those values, one can obtain a much more reliable estimate of the position of the CoP at that time. On the other hand, if the points are distant in time from each other (say e.g., 100 ms), then the movement of CoP during this time lapse is no longer negligible, and averaging those values will result in a less reliable estimate.

This idea leads to the notion of relevance interval (RI), which we define as follows:

**Definition 1** (**Revelance Interval**)**.**  *Let s be a time instant, Δ be a time duration and $T_{s}\left(\Delta \right)=\left\{t\in T,|t-s|\leq \Delta \right\}$ be a window centered around s. We denote by $t_{p},\cdots ,t_{q}$ the ordered times in $T_{s}\left(\Delta \right)$. Then, for every $t\in T_{s}\left(\Delta \right)$ we define $I_{T_{s}\left(\Delta \right)}\left(t\right)$ the relevance interval of t in the window:*

$$\begin{array}{cc} I_{T_{s}\left(\Delta \right)}\left(t_{i}\right) & =\frac{1}{2}\left(t_{i+1}-t_{i-1}\right)\;,\;\text{for}\;p<i<q\;\\ I_{T_{s}\left(\Delta \right)}\left(t_{p}\right) & =\frac{1}{2}\left(t_{p+1}-t_{p}\right)-\left(s-\Delta \right)\;\\ I_{T_{s}\left(\Delta \right)}\left(t_{q}\right) & =s+\Delta -\frac{1}{2}\left(t_{q}-t_{q-1}\right)\; \end{array}$$

This notion of RI allows us to define the Sliding Window Average with Relevance Interval Interpolation (SWARII) algorithm.

**Sliding Window Average with Relevance Interval Interpolation:** In this approach, the value at the desired time *s* is obtained using a Sliding Window and averaging all the values generated by the WBB in a temporal window centered around *s* of length Δ>0, by weighting every point with its RI.

$$X_{SWARII}\left(s\right)=\frac{1}{\Delta }\sum_{t\in T_{s}\left(\Delta \right)}x_{t}I_{T_{s}\left(\Delta \right)}\left(t\right)\;$$

where $X_{SWARII}\left(s\right)$ is the interpolated signal at time *s* and $T_{s}\left(\Delta \right)=\left\{t\in T,|t-s|\leq \Delta \right\}$. Note that $\sum_{t\in T_{s}\left(\Delta \right)}I_{T_{s}\left(\Delta \right)}\left(t\right)=\Delta$. The length Δ of the window controls the amount of smoothing of the estimation.

**Figure 2:**
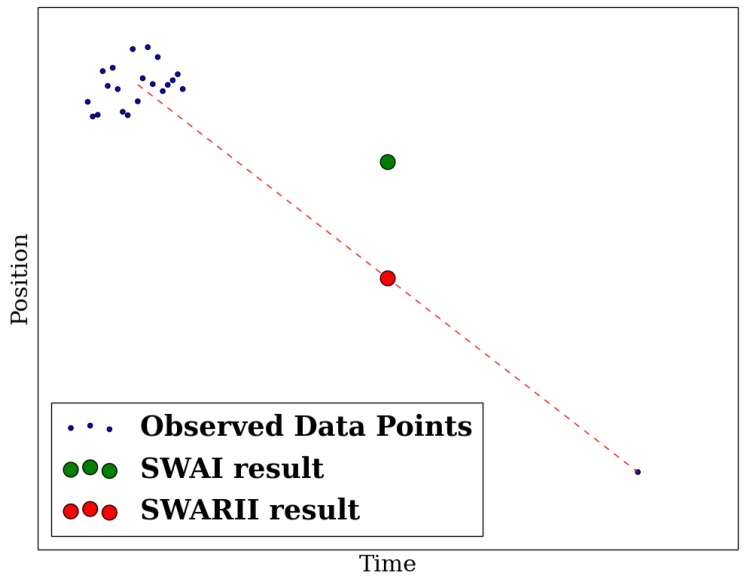
Illustration of the Sliding Window Average Interpolation (SWAI) and Sliding Window Average with Relevance Interval Interpolation (SWARII) methods. The data points are represented in blue, the result of SWAI in green, and the result of SWARII in red. Since the SWAI algorithm performs a uniform average, its result is strongly influenced by the cluster of points to the left and almost oblivious to the right point, while SWARII attributes an almost equal weight to the cluster to the left and to the point to the right.

The idea of the algorithm is that if some points are very close to each other in time, their individual RI will be small, but their total RI will be large. Alternatively, if a point is isolated in time, its individual RI will be large. The result of the SWARII approach is illustrated by Figure 2.

### 2.4. Simulated Data

In order to analyse the respective performances and relative merits of the algorithms presented in Section 2.2 and Section 2.3, we compare them on some simulated dataset (comparisons on real WBB acquisitions are performed in the next subsection). The signals (piecewise linear and sinusoidal) are generated as follows: first, the ground truth signal *f* is defined as:

$$\begin{array}{cc} f_{\text{linear}}\left(t\right) & =10\min(t-6\lfloor t/6\rfloor ,6\lceil t/6\rceil -t)\;\\ f_{\text{sine}}\left(t\right) & =10\sin(\pi t/3)\; \end{array}$$

Then to generate the time intervals $\delta_{i}$ between any two successive samples $t_{i+1}$ and $t_{i}$, we use a mixture of the absolute values of two independent Gaussian distributions, to represent the multi-regimes distribution of the acquisition frequency of the WBB:

$$t_{i+1}-t_{i}=\delta_{i}∼p_{i}\left|N\left(0.02,0.01\right)\right|+\left(1-p_{i}\right)\left|N\left(0.1,0.01\right)\right|\;$$

where $p_{i}∼B\left(0.5\right)$, i.e., is the realization of 0.5-mean independent Bernoulli random variable.

Finally, the noisy signal is generated by adding to every data point an independent Gaussian random variable of mean 0 and standard deviation 0.1:

$$x_{i}∼f\left(t_{i}\right)+N\left(0,0.1\right)\;$$

On each of the linear and sinusoidal datasets, we apply a resampling algorithm (PCI, LI, SWAI, SWARII or cubic splines) to obtain a uniform signal sampled at 25 Hz. With the PCI and LI methods the resampled signal is then filtered using a 4th order 12 Hz low pass Butterworth. No filter is applied to SWAI and SWARII, as doing averages over a window already denoises the signal, or to the cubic splines, which already includes a regularisation component. Let ${\hat{x}}_{1},\cdots ,{\hat{x}}_{N}$ be the resulting values at the resampled time instants $s_{1},\cdots ,s_{N}$. To derive the speed at those time instants, we use the formula:

$$\frac{d\hat{x}}{dt}\left(s_{i}\right)=\frac{{\hat{x}}_{i+1}-{\hat{x}}_{i-1}}{s_{i+1}-s_{i-1}}\;$$

and we compare the different results, using the root mean square error (RMSE) *R* defined as:

$$R\left(d\hat{x}/dt,df/dt\right)=\sqrt{\frac{1}{N}\sum_{i=1}^{N}{\left(\frac{d\hat{x}}{dt}\left(s_{i}\right)-\frac{df}{dt}\left(s_{i}\right)\right)}^{2}}\;$$

In addition to the speed, we also compare the resulting Approximate Entropy (ApEn) of the different resampling methods. ApEn is a temporal dynamic feature which encode the randomness of time series [20], and previous works using ApEn have shown promising results (see, e.g., [21,22,23]). To compute the ApEn, we used the following formula:

$$\text{ApEn}\left(\hat{x},m,r\right)=\phi_{m}^{r}\left(\hat{x}\right)-\phi_{m+1}^{r}\left(\hat{x}\right)$$

where

$$\phi_{m}^{r}\left(\hat{x}\right)=\frac{1}{n-m+1}\sum_{i=1}^{n-m+1}\ln\left(\frac{1}{n-m+1}\sum_{j=1}^{n-m+1}1(\max_{k=0,\ldots ,m-1}\left(\right|{\hat{x}}_{i+k}-{\hat{x}}_{j+k}\left|)>r\right)\right)$$

where ${\left({\hat{x}}_{i}\right)}_{i=1}^{n}$ is the resampled signal, ln is the natural logarithm and 1 is the characteristic function. Following the recommendation of [23], we used m=2, r=0.2σ, where *σ* is the standard deviation of the signal. The results are presented in Section 3.1.

### 2.5. Experimental Data

In order to assess the impact of the proposed methods for real practitioners, the following empirical experiments were performed.

**Inanimate weight:** The first experiment is a simple setup where we place a dead weight of 45 kg on the WBB. The recorded signal is then only pure noise, and the true speed of the object is known to be constantly zero. This forms an efficient test to measure the ability of the candidate methods to face both the noise and the variations in the sampling rate. The analysis is performed as in Section 2.4 and the results are presented in Section 3.2.

**Subject comparisons with the AMTI:** The second experiment is a comparison with the laboratory grade force platform AMTI on real subjects. The statokinesigrams of 10 individuals were repeatedly recorded in random order on both the AMTI and the WBB, such that each individual were measured three times on both devices. Those repeated acquisitions on each force platform permit a more statistically significant analysis: successive statokinesigrams of a single individual may present differences, and computing statistics on a small sample (here 3 statokinesigrams instead of 1) reduces this variance. Additionally, and to reduce this variability even further, we restricted this experiment to healthy subjects.

For the experiment, the individual was asked to remove his/her shoes, and to step on the platform. The feet were placed in the most comfortable position for the patient without exceeding the shoulder width. The individual was asked to stand in upright position, arms laying at the side, with open eyes, and to look at the opposing wall at eye level. The displacement of the CoP was recorded for 30 s.

The statokinesigrams of 10 individuals were repeatedly recorded in random order on both the AMTI and the WBB, such that each individual were measured three times on both devices. The random shuffling of the order of the acquisitions permits to reduce the bias in the equilibrium of the subjects during the recording session. For each statokinesigram, we computed scalar features on the instantaneous speed such as its mean, standard deviation and quantiles. Computing features based on derivatives has a crucial role in posturography, and good resampling algorithms should take a lot of care in this direction, see for example [13,24] for various features based on velocity and acceleration. We also computed the Approximated Entropy, where the signal from the AMTI was sub-sampled to 25 Hz, and m=2 and r=0.2σ as in the previous section.

The empirical distributions of the features over the 30 recordings on the AMTI are then compared to the distributions over the 30 recordings on the WBB, with the various resampling strategies. This experiments permits to quantify the relative performances of the different methods with respect to the similarity to the AMTI. The results are presented in Section 3.3.

**Method:** On both experiments and for all the four considered algorithms, the resampling frequency was set to 25 Hz. For SWAI and SWARII, the time window was set to 0.5 s. For the cubic spline, the value of lambda was chosen among a logarithmic grid ranging from 0.01 to $10^{5}$ with a 10-folds cross validation. The speed was computed as the norm of the 2D-speed vector as derived in the previous subsection.

## 3. Results

### 3.1. Toy Dataset

**Figure 3:**
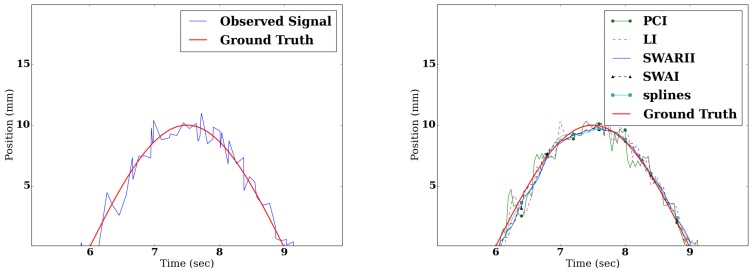
**Left**: comparison between the real sinusoidal signal (plain red) and observed signal (plain blue) detailed on a change of direction. **Right**: comparison between the reconstructed signals with Piecewise Constant Interpolation (PCI) (green dot), Linear Interpolation (LI) (dashed purple), SWAI (black triangle), SWARII (plain blue) and Cubic splines (cyan square).

**Figure 4:**
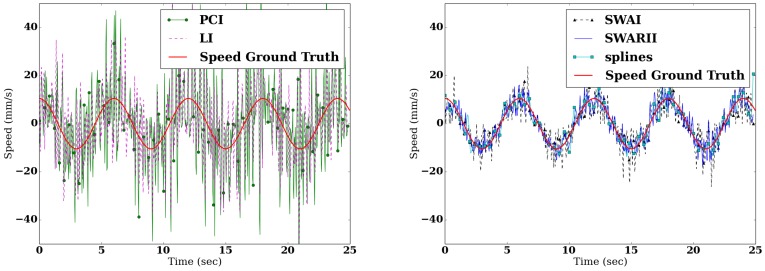
Comparison between the real sinusoidal speed (plain red) and the speed reconstructed from the signal with different methods: PCI (green dot), LI (dashed purple), SWAI (black triangle), SWARII (plain blue) and Cubic splines (cyan square).

Figure 3 shows the sinusoidal signal, during a change of direction, both observed and reconstructed. It is interesting to note that all the methods give reasonable estimates of the position of the CoP. However, when the reconstruction is differentiated to obtain the speed (Figure 4), their respective performances greatly differ. The same remarks hold true for the piecewise linear signal. The Root Mean Square Error (RMSE) of the difference between the real speed of the signal and the speed derived from the reconstructed signals are presented in Table 1 and Table 2. Table 3 shows the ApEn of the true signal, sampled at 25 Hz, compared to the ApEn of the reconstructed signals.

The PCI method gets the worse RMSE, both for speed and position. Since this method only use one point of the original signal for the interpolation, it reconstruction of the observed signal is heavily influenced by the noise, despite the low pass filter. Similarly, the LI method which uses two points of the original signal for the interpolation, is vastly outperformed by the other methods, while being better than the PCI. Those two methods are similar, and their poor performances are the results of two main drawbacks: first, those methods only use a very small amount of points to construct the interpolation, and as a consequence, they are much more prone to the noise-related errors, and second, the denoising part, a Butterworth low pass filter, can only be applied after the resampling. Similarly, due to the influence of the noise, those two methods largely overestimate the ApEn of the signal.

For speed estimation, SWAI produces average results, with the lowest standard deviation while SWARII obtains the best results, at the cost of a higher standard deviation, while regarding the Apen, it is interesting to note that those both methods underestimate the ApEn of the signal. This can be explained by the strong regularization part of those methods: both are based on the sliding window model, and in both cases the denoising is done at the same time as the resampling. The fact that SWARII outperforms SWAI gives credit to the intuition, developed in Section 2.3, that a careful use of the RI of the data points gives a significant advantage on noisy signal.

There are a few important facts to note about the Cubic Spline approach. First and foremost, the parameter *λ*, is difficult to choose and the performances of the algorithm varies greatly with it. A careful cross validation can select a nearly optimal lambda, at the cost of a significant increase of the run time of the algorithm and a greatly increased algorithmic complexity (for those experiments, the *λ* obtained by cross validation was 316.23). Second, while this method produces satisfying results, it is still outperformed by simpler and faster methods (like SWAI) while underestimating the ApEn of the signal (also like SWAI). In this experiment, this variability can be explained as follows: if a significant number of points is available in every interval, the cubic splines interpolation produces a nearly optimal interpolation; but in the case where few points are available, the performances greatly suffer.

### 3.2. Static Signal

**Figure 5:**
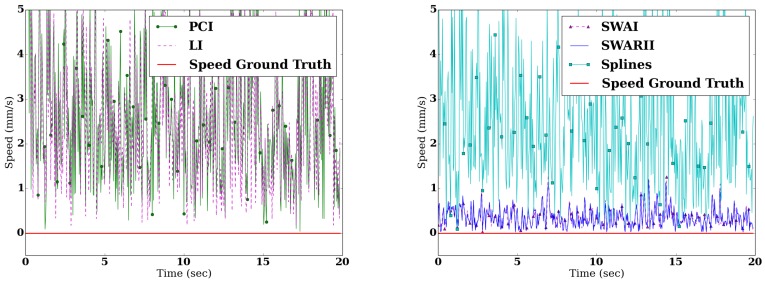
Comparison between the real speed of the inanimate object (plain red) and the speed reconstructed from the signal with different methods: PCI (green dot), LI (dashed purple), SWAI (black triangle) and SWARII (plain blue).

In this setting, an inanimate object was recorded during 20 s with the WBB. Hence, the speed and the ApEn are known to be zero, as the signal is constant. This allow for an easy evaluation of the different methods. Figure 5 shows the speed obtained by differentiating the signal reconstructed with the use of the different methods. The Root Mean Square Error (RMSE) of the difference between the real speed of the signal and the speed derived from the reconstructed signal are presented in Table 4, and the ApEn of the respective signals can be found in Table 3.

The performances of the respective algorithms confirm the result of the experiment—PCI and LI vastly overestimate the speed and the ApEn of the signal—on the toy dataset, expect for the cubic splines. Its clearly suboptimal results for speed estimation, and its poor estimation of the ApEn, are directly related to a poor choice of lambda (here λ=2.19). It is important to remember that *λ* is chosen by cross validation while trying to reconstruct the *signal*, not the speed. Thus, in this case, the lambda selected was clearly suboptimal. This highlights the main drawback of this method, namely the choice of *λ*.

### 3.3. WBB and AMTI Comparison

**Figure 6:**
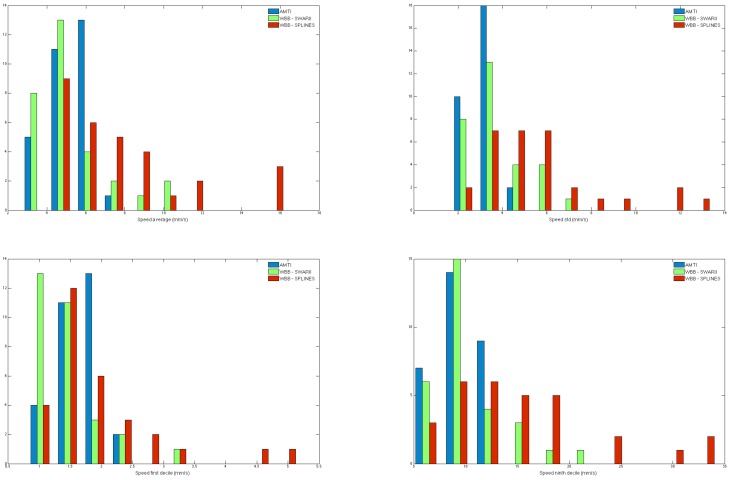
Comparison of the empirical distributions of scalar features using the AMTI platform (blue), using the WBB with Cubic Splines (red), and using the WBB with SWARII (green), for the average speed (**top left**), standard deviation (**top right**), first decile (**bottom left**), ninth decile (**bottom right**).

In this setting 30 statokinesigrams from 10 individuals were recorded on both the AMTI and the WBB. The signals from the AMTI were filtered using a Butterworth filter of order 4 and low pass cutoff at 12 Hz, and four scalar features of the instantaneous speed were computed on each signal: average, standard deviation, first decile and ninth decile. We then compared the distributions of the four scalar features obtained from the AMTI and the WBB processed by the five resampling methods: PCI, LI, SWAI, SWARII and Cubic splines. Figure 6 shows the empirical distributions of the four scalar features for the three cases : Cubic Splines, SWARII and AMTI. The distance between distributions obtained by the WBB using the different methods and the AMTI are quantified in Table 5 and Table 6 for the four resampling methods. The tables show the two-samples Kolmogorov-Smirnov test and the two-sided Wilcoxon rank sum test, which test if two empirical distributions are respectively drawn from the same distribution and from distributions with equal medians.

Those results confirm the previous conclusions. The SWAI and SWARII methods do perform significantly better than the others by giving distributions which are more similar to the AMTI. Indeed, the sliding window methods improve the results from each statistical test by several order of magnitudes against the PCI and LI methods. Note that the SWARII method improves only marginally the results over SWAI, but is mostly similar to the SWAI method in this experiment. Finally, the result of the the Cubic splines approach, while not being as good as the sliding windows methods, significantly outperforms the LI and PCI methods.

**Figure 7:**
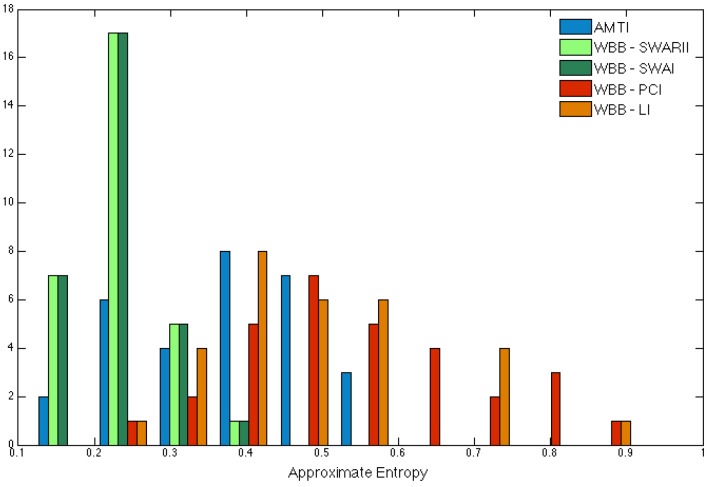
Comparison of the empirical distributions of Approximate Entropy using the AMTI platform (blue), using the WBB with SWARII (light green), with SWAI (dark green), with PCI (red) and with LI (orange).

We then computed the ApEn with same parameters as before, and the signal from the AMTI sub-sampled to 25 Hz, which corresponds to ApEn windows of 80 ms for m=2. The empirical distributions of the ApEn using the AMTI or the WBB with various methods are reported in Figure 7. The results using the WBB with cubic splines interpolation are not depicted on the figure, since cubic splines interpolation leads to a strongly underestimated ApEn due to the regularity of the interpolation with cross-validated parameter. This experiment with real subjects confirms the previous results with the synthetic signals, that is the ApEn is underestimated using SWAI or SWARII with a time window of 0.5 s, and overestimated using PCI or LI. Direction aside, the distances between the real ApEn and the reconstructed ApEn are similar for all the methods.

## 4. Discussion and Conclusions

As discussed in [12], the non uniform sampling frequency of the WBB puts significant restrictions when recording and studying signals such as the statokinesigrams. From our analysis, it is important to carefully chose the resampling algorithm, as while most methods give satisfying accuracy regarding the position of the CoP, and are comparable in terms of ApEn approximation, their performances greatly differ when the signal is differentiated. This is of paramount importance for the WBB, since the differentiation of the statokinesigrams is necessary to obtain speed or acceleration, which are key elements of the analysis in posturography (see, e.g., [13]).

To the extent of our knowledge, the CPI and LI methods are frequently used for resampling the WBB signal (see, e.g., [16,17]). As seen in Section 3, those methods lead to significant error when deriving the mean, standard deviation and quantiles of the speed, and are very inaccurate for computing the instantaneous speed. The use of a sliding window, as in SWAI or SWARII, gives significantly better results. Since SWAI is widely available in most data analysis software, it should always be preferred to CPI and LI when resampling data from the WBB.

The cubic splines approach, while providing solid theoretical guarantees, produces disappointing results in practice. This is largely due to the difficulty of choosing the parameter λ, and the fact that the quality of the signal of the WBB varies greatly during a record (e.g., different sampling rate).

It is worth noting that SWARII outperforms SWAI by a careful use of the relevance intervals of the data points. To achieve a better accuracy when acquiring a signal from the WBB, we highly recommend to use SWARII (An implementation of SWARII for Python 2.7 is available for download at: https://reine.cmla.ens-cachan.fr/j.audiffren/SWARII.git) or SWAI whenever possible as those methods require only one parameter, the length of the window.

It is the authors belief that more recent and state-of-the-art approaches to resampling can lead to better performances. However the complexity of those models, and the number of required parameters that need to be carefully tuned to prevent overfitting or flattening of the derivatives, make them difficult to use for non experts. Hence we have promoted in this paper the use of sliding window methods for their robustness as they rely on the proper calibration of only one simple parameter.

As for the calibration of the WBB using the method described by [12], the careful resampling of the data using an efficient method produces results which are significantly closer to the data obtained using the laboratory grade force platform AMTI.

However, and even if those methods improve the results, the WBB still lacks the accuracy, both temporal and spatial, recommended for Posturography. As [11] stated, the low signal to noise ratio—which is largely induced by the unshielded cables and electronics incapable of noise minimization—currently restricts the utilization of the WBB for clinical or research purposes. Hence, it should only be used for measuring statokinesigrams when laboratory grade force-platforms are unavailable and lower accuracy is acceptable.

## Figures and Tables

**Table 1:** Root Mean Square Error resulting from the approximation of the different resampling methods for the simulated linear signal.

	PCI	LI	SWAI	SWARRI	Cubic Splines
Position	1.13 (±0.06)	0.82 (±0.03)	0.58 (±0.03)	0.46 (±0.03)	0.57 (±0.09)
Speed	17.20 (±0.51)	12.40(±0.47)	7.79 (±0.15)	4.53 (±0.17)	5.90 (±2.00)

**Table 2:** Root Mean Square Error resulting from the approximation of the different resampling methods for the simulated sinusoidal signal.

	PCI	LI	SWAI	SWARRI	Cubic Splines
Position	1.04 (±0.04)	0.80 (±0.04)	0.46 (±0.03)	0.41 (±0.03)	0.47 (±0.06)
Speed	16.14 (±0.61)	11.97 (±0.45)	4.71 (±0.14)	3.01 (±0.19)	4.09 (±1.56)

**Table 3:** Approximate Entropy of the approximation of the different resampling methods.

	Ground Truth	PCI	LI	SWAI	SWARRI	Cubic Splines
Linear Signal	0.53	0.68	0.65	0.46	0.44	0.33
Sinusoidal Signal	0.44	0.75	0.62	0.35	0.34	0.34
Static Signal	0	1.75	1.63	0.35	0.35	1.80

**Table 4:** Root Mean Square Error of the speed approximation of the different methods.

	PCI	LI	SWAI	SWARII	Cubic Splines
Static Signal	2.75	2.6	0.69	0.50	3.19

**Table 5:** *p*-Values of two-samples Kolmogorov-Smirnov test comparing the distributions of the scalar features using the WBB against the AMTI with respect to the resampling method.

	PCI	LI	SWAI	SWARRI	Cubic Splines
Average speed	$10^{-12}$	$10^{-9}$	0.34	0.53	$10^{-3}$
Speed standard deviation	$10^{-8}$	$10^{-8}$	0.2	0.2	$10^{-6}$
Speed first decile	$10^{-13}$	$10^{-10}$	0.02	0.02	0.20
Speed ninth decile	$10^{-11}$	$10^{-9}$	0.34	0.34	$10^{-4}$

**Table 6:** *p*-Values of two-sided Wilcoxon rank sum test comparing the distributions of the scalar features using the WBB against the AMTI with respect to the resampling method.

	PCI	LI	SWAI	SWARRI	Cubic Splines
Average speed	$10^{-10}$	$10^{-9}$	0.46	0.47	$10^{-3}$
Speed standard deviation	$10^{-9}$	$10^{-8}$	0.36	0.44	$10^{-6}$
Speed first decile	$10^{-10}$	$10^{-10}$	0.03	0.04	0.48
Speed ninth decile	$10^{-10}$	$10^{-9}$	0.76	0.77	$10^{-4}$

## Abbreviations

The following abbreviations are used in this manuscript:

WBB: Wii Balance Board
CoP: Center of Pressure
RI: Relevance interval
PCI: Piecewise Constant Interpolation
LI: Linear Interpolation
SWAI: Sliding Windows Average Interpolation
SWARII: Sliding Windows Average with Relevance Interval Interpolation
ApEn: Approximate Entropy
